# Nailing of diaphyseal ulna fractures in adults—biomechanical evaluation of a novel implant in comparison with locked plating

**DOI:** 10.1186/s13018-020-01656-z

**Published:** 2020-04-20

**Authors:** Johannes Christof Hopf, Dorothea Mehler, Tobias Eckhard Nowak, Dominik Gruszka, Daniel Wagner, Pol Maria Rommens

**Affiliations:** 1 Department of Orthopedics and Trauma Surgery, University Medical Center, Langenbeckstraße 1, 55131 Mainz, Germany; 2 Mainz, Germany

**Keywords:** Nailing, Ulna shaft, Biomechanical study

## Abstract

**Background:**

Adult forearm fractures require surgical treatment in most cases. Open reduction and internal fixation with plate osteosynthesis is the therapy of choice. Intramedullary fixation offers several advantages compared to plate fixation but is not routinely used. The aim of our study was to compare a newly designed ulna nail with angular stable plating in a biomechanical testing setup of an ulna shaft fracture with a diaphyseal defect.

**Methods:**

Ten pairs of sawbones with a defect osteotomy of the ulna shaft (OTA 2U2C3) were fixed with an interlocked nail or locked plate osteosynthesis. The constructs were tested under four-point bending, torsional loading and axial loading in a servo-pneumatic testing machine to compare the stiffness of both stabilization methods.

**Results:**

The nail constructs show lower yet sufficient bending stiffness (62.25 ± 6.64 N/mm) compared to the plate constructs (71.2 ± 5.98 N/mm, *p* = 0.005). The torsional loading test shows superior stiffness of the plate constructs (0.24 ± 0.03 Nm/deg vs. 0.1 ± 0.01 Nm/deg; *p* < 0.001), while the axial loading shows superior stiffness of the nail constructs (1028.9 ± 402.1 N/mm vs. 343.9 ± 112.6 N/mm; *p* < 0.001).

**Conclusions:**

Intramedullary nailing of ulna shaft fractures obtains sufficient but lower stability in bending and torsional loading when compared to rigid angular stable plating and could be an alternative technique to plate fixation. The lower stability and the closed stabilization technique allow for a rapid periosteal healing, which is not present in stiffer constructs.

## Introduction

In adult diaphyseal forearm fractures, precise restoration of the anatomy is crucial to achieve bony union and good functional results. Therefore, most adult diaphyseal forearm fractures require surgical treatment [1]. Several surgical methods have been described for their treatment including plate-screw osteosynthesis and intramedullary nailing [2]. Open reduction and plate osteosynthesis of forearm fractures is an accepted treatment option and showed good functional results since many years [3]. Comminuted or segmental forearm fractures increase the risk for infection and non-union when treated with open reduction and plate osteosynthesis. Intramedullary fixation is less often used in adult forearm fractures, although they offer several advantages compared to plate fixation as less periosteal stripping, preservation of the fracture hematoma, and biomechanical advantages of a central load-bearing implant [1, 4–6]. This could be explained with a challenging implantation process with difficulties in restoration of forearm geometry. Also, the risk of neurovascular injuries by malpositioned locking screws can lead to severe complications.

Comparable functional outcomes of nail and plate fixation were described in several clinical studies [7–9]. Nailing of the ulna combined with plating of the radius is described as an acceptable hybrid fixation method for both-bone forearm fractures [10]. A combined biomechanical and clinical study comparing plating and nailing techniques in adult forearm fractures favored the combination of ulna nailing and radius plating because of better functional outcomes, fewer complications, and good biomechanical results compared to other osteosynthetic techniques (both-bone nailing, both-bone plating, ulna plating, and radius nailing) [11].

Biomechanical principles established by the AO/ASIF (Arbeitsgemeinschaft für Osteosynthesefragen/Association for the Study of Internal Fixation) postulated that a certain amount of strain by a load-sharing implant like an intramedullary nail is favorable for periosteal bony healing with callus. This principle especially applies in comminuted fractures and is used successfully in other long bone fractures [12].

The aim of our study was to compare the biomechanical characteristics of a newly designed, anatomically shaped ulna nail with angular stable plating in a biomechanical test setup of an unstable ulna shaft fracture.

## Materials and methods

This study is a biomechanical comparison of a newly developed ulna nail and a standard angular stable plate in an ulna shaft fracture model using sawbones. We performed a four-point bending, a torsional loading, and an axial loading test.

### Bones and fracture type

We used ten pairs of large left 4th generation composite sawbones (#3426, Sawbones® Pacific Research Laboratories, Vashon Island, USA) for the biomechanical testing. We simulated a segmental ulna shaft fracture (OTA 2U2C3 (Orthopaedic Trauma Association)) with high grade of instability by creating a standardized shaft osteotomy with a 10-mm gap.

### Implants

**Fig. 1:**
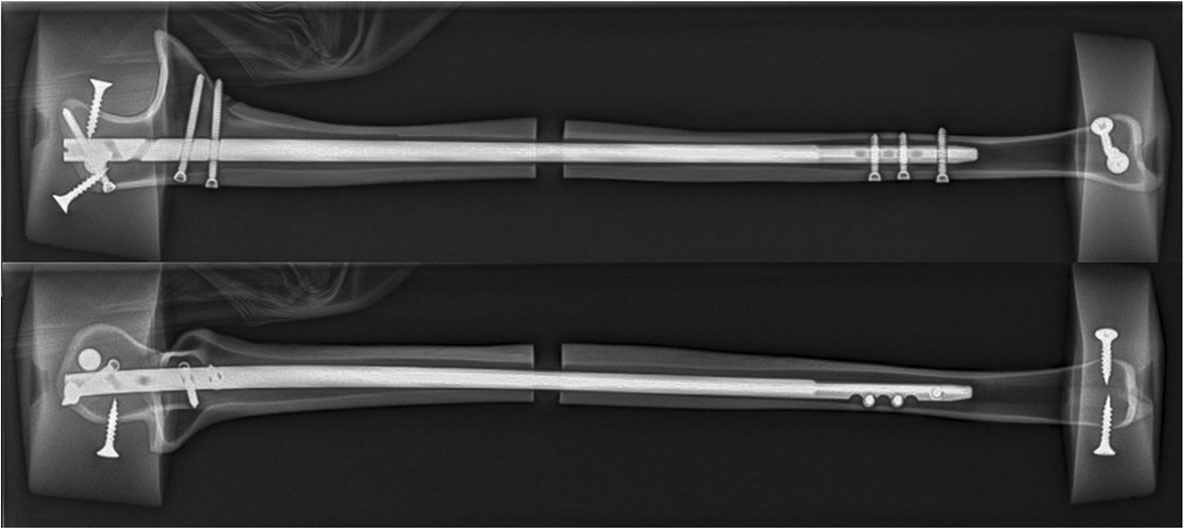
X-ray in anterior-posterior and lateral view of a left nail with inserted locking screws after potting in PMMA cement

The newly developed ulna nail was developed in a cooperation agreement between the authors and MEDIN a.s. (Nove Mesto na Morave, Czech Republic). Prior to the determination of the characteristics of the new nail, a CT-graphic study of the ulna shaft was performed. The feasibility of implantation of the newly designed nail was checked in cadaveric tests. We used ten nails made of forged titanium (Ti-6Al-4 V ELI) with a diameter of 6 mm in the proximal part and 5 mm in the mid-shaft and distal part. The length of the nail was 230 mm with a radial bending within the proximal 120 mm with a radius of 735 mm (9° radial bending). The nail offers two retrograde locking options in the proximal part placed towards the tip of the olecranon and three metaphyseal locking options placed into the coronoid process. The locking is done with 2.7 mm locking screws with threaded heads for stable fixation in the first cortex. All proximal and metaphyseal locking screws are inserted through a targeting device. The angulation of the metaphyseal screws prevents an intraarticular screw positioning into the proximal radio-ulnar joint. The distal locking is done free-hand, ideally with a radiolucent drive in anterior-posterior direction. The nail offers one locking hole and notches proximal to the hole for easier locking. Figure 1 shows an X-ray in anterior-posterior view and lateral view of the nail with inserted locking screws and after potting in polymethylmethacrylate cement. For the second group, ten angular stable 3.0 mm plates with a length of 84 mm (7 holes) were used.

### Implantation process

**Fig. 2:**
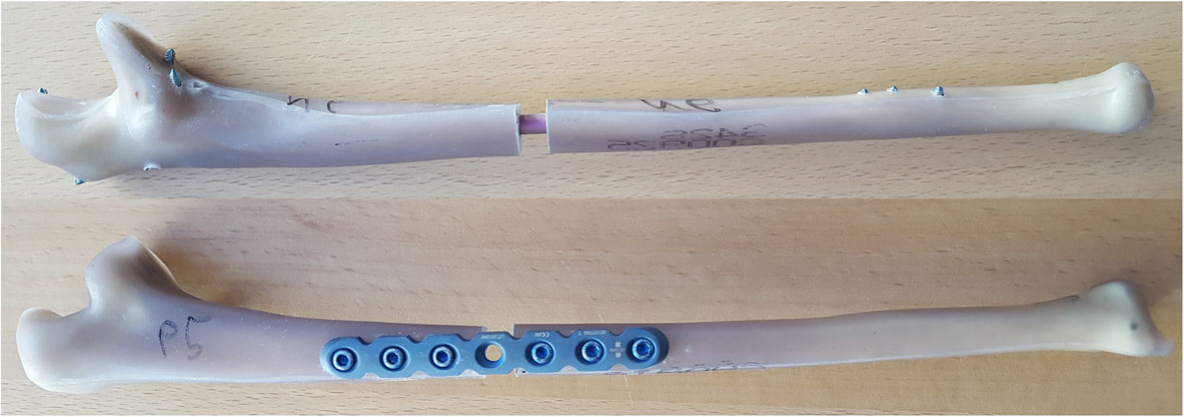
Nail und plate construct after osteosynthesis in lateral view

The nails were implanted in a standardized technique with prior reaming of the intramedullary canal with hand reamers. The canal was reamed up to 7 mm in the proximal part and 6 mm in the distal part to allow easy insertion of the nail. All proximal and metaphyseal locking options were used in the nail. The distal locking was done under image intensifier with three screws, one through the hole and two into the notches. The plates were fixated with three 3.5 mm locking screws proximal and distal to the osteotomy (6 cortices on each side) on the dorso-ulnar side of the ulna. Figure 2 shows both constructs after the implantation process. All constructs underwent X-rays in two planes to verify correct implant and screw positions.

### Testing setup

A servo-pneumatic testing machine (SincoTEC, Clausthal-Zellerfeld, Germany) was used for the biomechanical tests. The constructs were loaded to accomplish a linear elastic deformation; testing of load-to-failure was not intended. We measured the stiffness values with an established method using the applied forces and torques and the resulting movements [13].

**Fig. 3:**
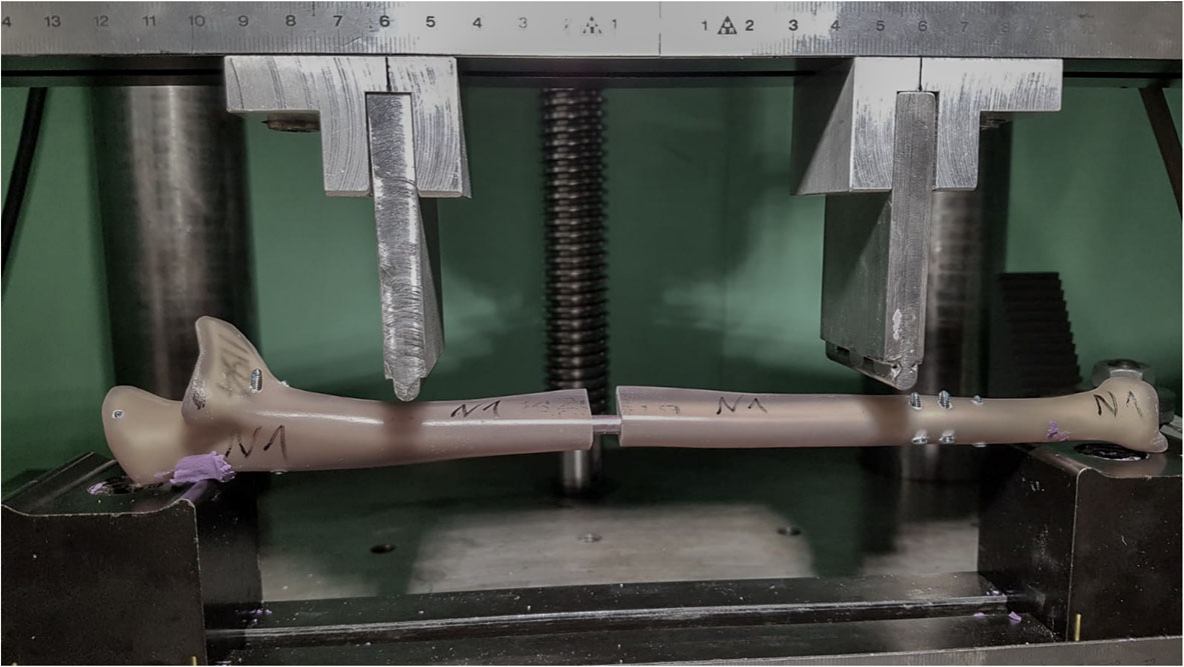
Four-point bending test setup with mounted nail construct

For the four-point bending test, the constructs were placed in the testing machine with a preload of 10 N. Then, a bending moment of 7.5 Nm was applied to the samples in an anterior-posterior direction. Six load changes were performed with a frequency of 0.1 Hz. For measurement of the bending stiffness, the last three load changes were used. Figure 3 shows the four-point bending testing setup with clamped nail construct.

After the four-point bending test, the constructs were potted in polymethylmethacrylate cement (PMMA) with added screws to secure the fixation and fixed in the testing machine at both ends. The torsional loading was done torque-controlled with a torque of 2 Nm. Six load changes were performed with a frequency of 0.05 Hz. Again, three pre-cycles and three measurement cycles were performed to determine torsional stiffness.

Finally all constructs were loaded under axial compression with a force of 250 N and a preload of 10 N. Six load changes with a frequency of 0.1 Hz were performed. The last three load changes were used for the measurement of the axial stiffness.

**Fig. 4:**
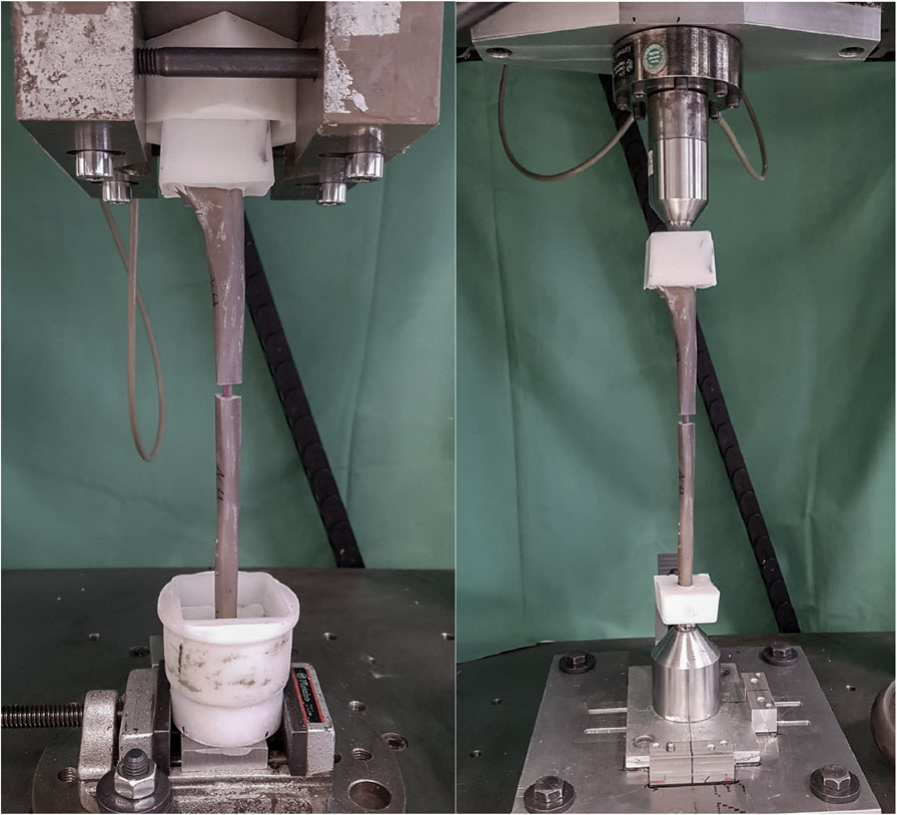
Test setups for the torsional and axial loading with mounted nail construct

The analysis of the four-point bending and axial loading was done using the force-displacement-diagram. The torsional stiffness was determined from the values of the torque-angle-diagram. Figure 4 shows the test setup for the torsional and axial loading.

### Data analysis and statistics

With an alpha level set at 0.05, the sample size of ten pairs of sawbones was calculated for a power (1–β) of 0.8 and an effect size of 1.4. For the statistical test, a Mann-Whitney Rank Sum test for independent samples was used with the statistical software SigmaStat (Systat Software GmbH, Erkrath, Germany).

## Results

No implant failure occurred in any construct. We detected no macroscopic implant loosening in both groups after test completion. Table 1 shows the results of the four-point bending, torsional loading, and axial loading tests. All constructs showed a linear elastic behavior in our testing setup; no plastic deformation occurred.

**Table 1:** Results of the four-point bending, torsional loading, and axial loading tests and *p* value after Mann-Whitney Rank Sum test

Parameters	Nail	Plate	***p*** value
Four-point bending [Nm/deg]	62.25 ± 6.64	71.2 ± 5.98	*p* = 0.005
Torsional loading [Nm/deg]	0.1 ± 0.01	0.24 ± 0.03	*p* < 0.001
Axial loading [N/mm]	1028.9 ± 402.1	343.9 ± 112.6	*p* < 0.001

### Four-point bending

**Fig. 5:**
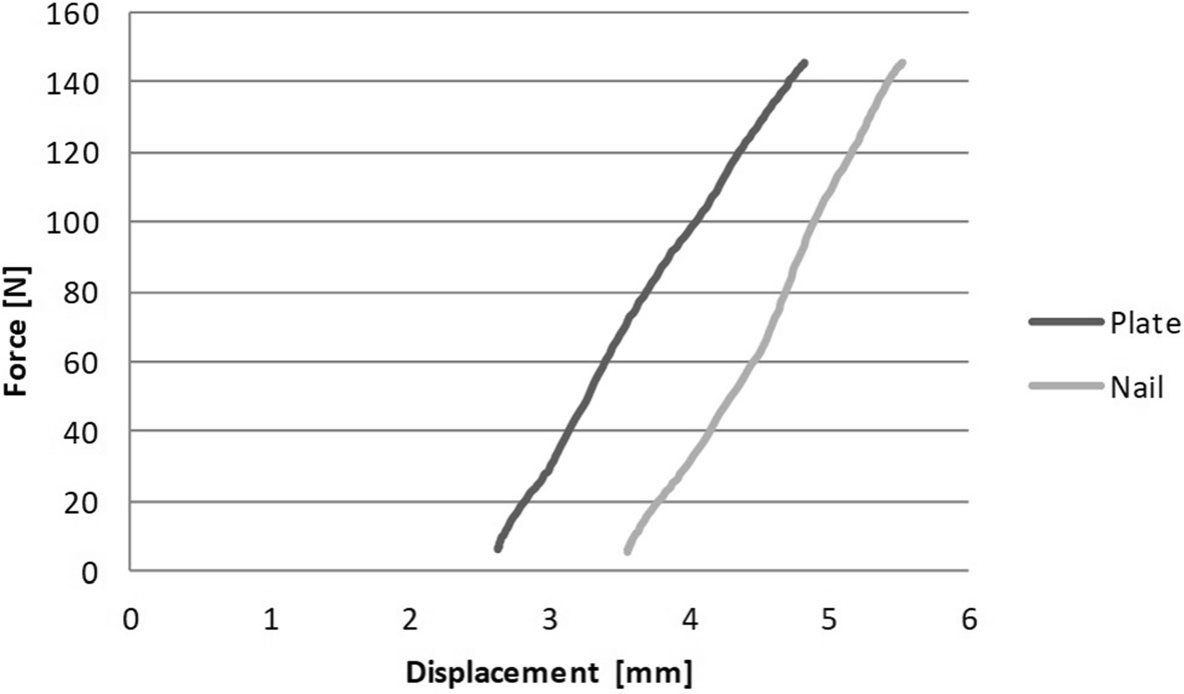
Force-displacement-diagram of pair four of the constructs

The plate constructs showed a higher bending stiffness (71.2 ± 5.98 N/mm) compared to the nail constructs (62.25 ± 6.64 N/mm) in anterior-posterior direction (*p* = 0.005). The average bending stiffness of the nail constructs amounts approximately 87% of the plate constructs. Figure 5 shows the force-displacement-diagram of pair 4 of the constructs for four-point bending.

### Torsional loading

The plate constructs showed higher torsional stiffness (0.24 ± 0.03 Nm/deg) compared to the nail constructs (0.1 ± 0.01 Nm/deg; *p* < 0.001). The torsional stiffness of the nail constructs amounts approximately 42% of the plate constructs.

### Axial loading

For the testing under axial loading, intramedullary implants have a concept-related advantage compared to extramedullary implants. The nail constructs had a significantly higher axial stiffness (1028.9 ± 402.1 N/mm) compared to the plate constructs (343.9 ± 112.6 N/mm; *p* < 0.001). The axial stiffness of the plate constructs amounts about one third compared to the nail constructs.

## Discussion

Since 1958, the basic AO principles for fracture treatment are worldwide accepted [14]. Rigid fixation under fracture compression leads to primary bone healing without callus formation. The fixation of comminuted fractures usually does not allow anatomic reduction and compression across the fracture. These fractures have the best chances of bony union with an osteosynthetic fixation, which allows a certain amount of strain using more working length [15]. Our results show significant higher bending and torsional stiffness of the plate constructs. Load-sharing devices like bridge plates or intramedullary nails allow a controlled amount of strain, which stimulates callus formation and secondary bone healing [1].

Intramedullary fixation of ulna fractures is a well-established technique in children [16]. For adolescents, the clinical results of intramedullary forearm fixation are varying. In comparison to elastic stable intramedullary nailing, good clinical results could be published for locked intramedullary implants [17]. Some authors described an increased complication rate for intramedullary techniques compared to the pediatric population [18, 19]. Especially the risk of non-union increases with increasing age due to insufficient stability of unlocked intramedullary implants [20, 21]. The development and use of locked intramedullary implants showed promising clinical results in the non-pediatric population with diaphyseal forearm fractures in several studies [22–25].

Intramedullary locked nailing offers fracture stabilization with an intended amount of strain combined with other relevant advantages of a closed surgical technique like preservation of the fracture hematoma and less impairment of the periosteal blood supply at the fracture site. We postulate that the characteristics of the newly designed long ulna nail are advantageous for uneventful bone healing, despite the lower stability in bending and rotation. With this biomechanical study, we surely cannot proove this hypothesis, and further clinical studies are necessary for evaluation. Until today, indications for ulna nailing are often restricted to special cases like pathologic fractures or revision surgery [26, 27]. Historically, especially drawbacks like immature implants and techniques, which resulted in insufficient stability and a high complication rate, prevented an enforcement of intramedullary nailing of ulna fractures [28]. With further development of the implants and techniques, like the introduction of locked implants, the clinical results improved, and fracture union could be achieved in a vast majority of cases [29–31].

Jones et al. described a biomechanical comparison of plate fixation and unlocked intramedullary rods in 1995 [32]. The torsional stiffness of the nail constructs (2.23% of intact forearm) were significantly lower compared to the plate constructs (83.4% of intact forearm) in this study [32]. In a recent biomechanical study, both-bone forearm nailing showed slightly lower stability compared to both-bone plate fixation [11]. The combination of ulna nailing and radius plating showed good biomechanical stability with the lowest complication rate and best clinical outcome in a clinical examination [11]. For the mentioned hybrid fixation of adult forearm fractures, Lee et al. showed good clinical results as well [33]. We expected higher torsional stiffness of the plate constructs in our study because of the lower distance of the locking screws to the osteotomy gap. Due to the primary bone healing without callus formation and due to periosteal damage by the plate, a relevant risk of refracture after plate removal is described [34]. We do not expect the same rate of refractures after nailing like shown for clavicle fractures [30, 35]. Despite the mentioned improvements of the implants and good biomechanical and clinical results, locked ulna nailing is not a frequently used treatment option. Our newly developed nail offers an anatomically preshaped design and several locking options with the possibility of angular stable screw fixation in the first cortex. We hypothesize to extend the indications for locked ulna nailing with this novel implant in particular for comminuted and segmental fracture of the ulna shaft.

Limitations of our study include the use of artificial bones instead of cadaveric bones and the fact that it is a biomechanical test, which can only approximate physiological conditions. Fresh-frozen cadaveric bones are closer to in vivo conditions in most of their biomechanical characteristics. On the other hand, differences in bony density or bone geometry of cadaveric bones can be a restriction as well [36]. Also, comparable biomechanical characteristics of composite bones compared to cadaveric bones with less variability in bone quality are described [37]. Because of the mentioned limitations and the fact that biomechanical studies cannot reproduce the behavior of soft tissues and physiological bone healing conditions, our results have to be interpreted carefully and cannot be transferred uncritical into the clinical setting.

In summary, our biomechanical study shows lower stability of the newly developed ulnar nail in bending and torsion than compared to angular stable plate osteosynthesis. The higher elasticity of the construct allows a certain amount of strain at the fracture site. Moreover, fracture hematoma and periosteal blood supply are preserved, which support callus formation and bony healing. These theoretical considerations must be evaluated in further clinical examinations to verify advantages and disadvantages of the new implant and its surgical technique in clinical practice.

## Conclusion

In this biomechanical study, we compared a newly designed intramedullary nail and an angular stable plate in an ulnar defect fracture under bending, torsional, and axial load. Our results show superior biomechanical stability under four-point bending and torsional loading in the plate group. In consideration of the strain theory for diaphyseal fracture healing, the elasticity of the nail construct may promote secondary bone healing. This hypothesis must be proved by clinical examinations. We conclude that this technique could be an alternative method for the treatment of unstable ulna fractures.

## Data Availability

The datasets used and/or analyzed during the current study are available from the corresponding author on reasonable request.

## Abbreviations

OTA: Orthopaedic Trauma Association
AO: Arbeitsgemeinschaft für Osteosynthesefragen
ASIF: Association for the Study of Internal Fixation
